# Dietary Intake Quality Is Affected by Knowledge and Dietary Intake Frequency among Pregnant Women in Muntinlupa, Philippines: A Cross-Sectional Study

**DOI:** 10.3390/ijerph182312306

**Published:** 2021-11-23

**Authors:** Tadashi Yamashita, Ramon Emilio Daniel Roces, Cecilia Ladines-Llave, Maria Teresa Reyes Tuliao, Mary Wanjira Kamau, Chika Yamada, Yuko Tanaka, Kyoko Shimazawa, Saori Iwamoto, Hiroya Matsuo

**Affiliations:** 1 Faculty of Nursing, Kobe City College of Nursing, 3-4 Gakuennishi-machi, Nishi-ku, Hyogo, Kobe 651-2103, Japan; shimazawa@tr.kobe-ccn.ac.jp (K.S.); iwamoto@kobe-ccn.ac.jp (S.I.); 2 Ospital ng Muntinlupa, Civic Drive, Filinvest Corporate City, Alabang, Muntinlupa 1771, Philippines; redroces@yahoo.com (R.E.D.R.); caladinesllave@gmail.com (C.L.-L.); 3 City Health Office, City Government of Muntinlupa, Centennial Avenue, Tunasan, Muntinlupa 1770, Philippines; materesatuliao63@gmail.com; 4 School of Nursing Sciences, University of Nairobi, P.O. Box 19676, Nairobi 00202, Kenya; kwanjira@uonbi.ac.ke; 5 Department of Environmental Coexistence, Center for Southeast Asian Studies, Kyoto University, 46 Shimoadachi-cho, Yoshida Sakyo-ku, Kyoto 606-8501, Japan; chika128@gmail.com; 6 Department of School Health Sciences, Institute of Biomedical Sciences, Tokushima University, 2-24 Shinku-racho, Tokushima 770-8501, Japan; yukon-tanaka@tokushima-u.ac.jp; 7 Department of Nursing, Osaka Shin-ai College, 6-2-28 Tsurumi, Tsurumi-ku, Osaka 538-0053, Japan; matsuohyroya0711@gmail.com

**Keywords:** food diversity, dietary status, pregnant women, knowledge, food frequency

## Abstract

Improving the nutrition of pregnant women is essential in reducing maternal and child mortality, which is one of the global nutritional goals of 2025. This study evaluated the factors related to the quality of dietary intake among pregnant women in Muntinlupa, Philippines. We conducted a cross-sectional study of 280 pregnant women at a hospital in Muntinlupa from March 2019 to August 2019 using questionnaires. After the primary aggregation, multivariate logistic regression analysis was used to identify factors associated with the quality of dietary intake in pregnant women. Approximately half of the women (46.4%, *n* = 130) had a low dietary diversity during pregnancy. Less than 30% of the respondents consumed beans, soybean products, and nuts. In the logistic regression analysis, poor maternal knowledge of nutritional sources to prevent anemia (odds ratio (OR) 4.25, 95% confidence interval (CI) 1.47–12.32, *p* = 0.01) and less frequent meal consumption (OR 2.15, 95% CI 1.08–4.29, *p* = 0.03) were significantly associated with poor dietary diversity. Our findings are crucial because they suggest that increasing the knowledge of pregnant women about good nutrition and ensuring that dietary intake is frequent and adequate through antenatal care can improve the nutrition of pregnant women.

## 1. Introduction

Micronutrient deficiency causes intrauterine fetal stunting, labor disorders, neonatal and childhood health abnormalities, and decreased quality of life of the mother during pregnancy and after childbirth [1,2,3,4,5,6,7,8]. Iron deficiency anemia, one of the most prevalent micronutrient deficiencies, is the most common cause of indirect obstetric death, and women who develop this condition during pregnancy are at risk of excessive bleeding, sepsis, and death during childbirth [9,10,11,12,13]. The World Health Organization aims to reduce anemia and low birth weight by 50% and 30%, respectively, by 2025 [14]. Past studies have shown that dietary diversity is significantly associated with nutritional value in the Philippines [15], Vietnam, Mali, and Peru [16,17,18]. This means that improving dietary quality is important to prevent anemia, especially in low- and middle-income countries.

Improvement in health and well-being, including maternal health, is the third sustainable development goal of the United Nations Development Program for the Philippines [19]. According to the results of a national nutrition survey conducted in the Philippines in 2008, 43% of pregnant women suffer from anemia; therefore, this is a major health problem in the country [20]. In addition, the current nutrition guidelines in the Philippines reflect the results of the 2008 National Nutrition and Health Survey, in which the importance of eating a more diverse diet during pregnancy was emphasized [21]. According to the 2008 results of the National Nutrition Survey, 26.3% or about one in every four pregnant Filipino women are nutritionally at risk [22]. Currently, the nutritional status of pregnant women is being improved under the Philippine Health and Nutrition Policy [23], although it has not been successful enough to improve the quality of dietary intake of pregnant women.

A joint team of Filipino and Japanese researchers has been implementing the project, “Primary Prevention of Iron Deficiency Anemia During Pregnancy” since 2019, and our study is a part of this project. The researchers of this project comprise Filipino doctors, Japanese doctors, and Japanese health science experts. Through this project, a study on the factors affecting iron intake in pregnant women in the Philippines has been reported [24], and through the current study, we aimed to focus on the quality of dietary intake of pregnant women. Therefore, we investigated the socioeconomic status of pregnant women and their dietary intake during pregnancy and analyzed the factors that affect the quality of their diet. The results of this study contribute to improving the quality of life of pregnant women in the Philippines, which in turn contributes to improving the health of fetuses and pregnant women.

## 2. Materials and Methods

### 2.1. Design and Sampling

A cross-sectional study was conducted in the department that promotes women’s health at the Hospital of Muntinlupa. Our targets were residents of different economic levels; therefore, we chose the Hospital of Muntinlupa, which is used by all residents in Muntinlupa. The data were collected between March and August 2019. The city of Muntinlupa consists of nine districts and is located south of Manila and is a highly urbanized city. The city has a total population of 504,509 in 2015. Muntinlupa has a GDP per capita growth of 5.0% (2017) and is experiencing rapid economic growth. On the other hand, there are a certain number of poor people who live in dangerous areas such as riverbanks and are vulnerable to disasters such as floods and fires.

Muntinlupa City has three government hospitals and eight private hospitals, with one health center in each district of the city. The Hospital of Muntinlupa is one of the government hospitals that provide medical services to low-income earners. The hospital is also one of the largest hospitals in the region, serving more than 1000 births annually. In addition, in the women’s health promotion departments of the hospitals, doctors and nurses provide vaccinations, health consultations, and treatment advice as part of preventive healthcare to women living in the community.

### 2.2. Sample Size and Sampling Procedure

The sample size required for this study was estimated by calculating the proportion of a single population, based on the following assumptions: a 33.0% rate of poor dietary diversity among pregnant women in the Philippines [25], a 95% confidence interval (CI), and a 5% acceptable margin of error. The calculated sample size was 240, and the final sample size was set to 267 to account for the 10% non-response rate. Using a systematic random sampling method, pregnant women who are eligible for prenatal care were selected from the 900 pregnant women who underwent their first or second prenatal care at the Hospital of Muntinlupa. All pregnant women, regardless of their gestation period were included, and then, pregnant women with serious illness or obstetric complications were excluded from the study. Eventually, 280 participants were included in this study. The sampling rate (K) was *N*/*n* = 900/280 = 3.2. We adopted a lottery method to identify the respondents for the first interview, and for the subsequent interviews, every third pregnant woman was selected.

### 2.3. Study Population and Sampling

The study population consisted of pregnant women between the ages of 18 and 45 years who lived around the Hospital of Muntinlupa and visited the hospital for prenatal care. The participants received antenatal care, immediately after which the questionnaires, written in Tagalog (the national language of the Philippines), were administered by a trained Filipino research assistant.

### 2.4. Data Quality and Processing

The questionnaire comprised 38 closed-ended questions that were created and pretested for use in this study. The questions were categorized as follows: sociodemographic characteristics (*n* = 6), food diversity (*n* = 11), current dietary practices (*n* = 7), maternal knowledge of major signs and symptoms of anemia (*n* = 7), and maternal knowledge of food sources that increase blood iron levels (*n* = 7). While food diversity has been assessed to determine the overall diet quality in previous studies [26,27], in this study, it was investigated as a measure of the quality of dietary intake using the 11-item Food Diversity Score Kyoto (FDSK-11) [28,29,30]. The reason why we used the FDSK-11 was that pregnant women in the Philippines were found to be more likely to respond to FDSK-11 from our preliminary investigation. The FDSK-11 is a screening tool for evaluating dietary quality based on the frequency of food intake of 11 food groups (grains, potatoes, meat, fish and shellfish, eggs, milk and dairy products, vegetables, seaweed, beans and soybean products, nuts, and fruits) over a week during the last six months [28]. The main outcome variable in this study was the FDSK-11 score, ranging between 0 and 11 points. The higher the score, the greater the food diversity, which indicates high quality of the dietary intake of the participants. The mean FDSK-11 score was 8.54 (standard deviation [SD], 1.80). Thus, respondents with a score of less than 9 were classified as having inadequate dietary diversity, while those with a score of 9 or higher were classified as having adequate dietary diversity. Dietary diversity was coded as 1 for those who showed inadequate dietary diversity and 0 for those who exhibited sufficient dietary diversity.

To ensure the reliability of the study, the test–retest method, in which measurements were taken at the beginning and two weeks later, was adopted. We evaluated the test results using Cohen’s kappa statistic to measure the agreement between the ratings of the two tests. After comparison, the questionnaire was considered reliable because the kappa values for all questions were above 0.7, and all the questions were retained. To ensure relevance, the questionnaires were shared, and their contents were discussed with specialists (doctors, nutritionists, nurses, and midwives) in the local health department of Muntinlupa City.

### 2.5. Data Analyses

The data were analyzed using the same method as in the previous study [24]. In this study, participants who did not answer questions about age, pregnancy, and diet were excluded from the analysis. To assess the level of knowledge regarding daily food intake in decreasing anemia incidence during pregnancy, respondents were asked two questions [31]: (1) major signs and symptoms of anemia during pregnancy and (2) food sources that increase blood iron levels in the body during pregnancy. The participant’s level of knowledge was calculated by adding the knowledge scores of the seven items related to the symptoms of anemia (“Feels weak,” “Looks pale,” “Palpitations,” “Headaches,” “Dizziness,” “Tiredness and easily fatigued,” and “Swollen legs”) and those related to the seven food items that increase blood iron levels in the body during pregnancy (“Liver”; “Red meat, e.g., beef”; “White meat, e.g., chicken and fish”; “Dark-green leafy vegetables”; “Whole-grain cereals, e.g., maize and sorghum”; “Legumes, e.g., beans and peas”; and “Eggs”). In the calculation, the correct answer score for each item was set to 1 and the incorrect answer score was set to 0. Participants with scores above the median and below the median were categorized into the highly knowledgeable group and slightly knowledgeable group, respectively, based on the knowledge of symptoms of anemia and food sources that increase blood iron levels in the body during pregnancy. To test the multicollinearity between the independent variables, we performed collinearity diagnosis and calculated the variance inflation factor for each variable. We then performed bivariate logistic regression analysis to evaluate the unadjusted association between the dependent variable and each independent variable. However, to maintain the reliability of the analysis, the number of independent variables used in the test was limited. Then, significant variables from which (*p* < 0.01) were incorporated into the multivariate logistic regression analysis to identify factors related to dietary diversity among the participants. All the data in this study were analyzed using the Statistical Package for the Social Sciences, version 26.0 (IBM Corp., Armonk, NY, USA).

## 3. Results

Table 1 shows the characteristics of the study participants. In total, 290 women participated, out of whom 10 were excluded because of missing data, and we ultimately analyzed the data of 280 women. The average age of study participants was 28.3 (SD, 6.5) years, and more than half (64.4%, *n* = 177) of them were unemployed. A majority (88.2%, *n* = 246) of the participants had an above secondary school education. Considering average household monthly income, the number of participants (59.6%, *n* = 159) who earned less than 9999 Philippine pesos was higher than those who earned above 10,000 Philippine pesos. Compared with primiparous women, a little more than half of them were multiparous (59.3%, *n* = 166). Furthermore, most of the participants were in their third trimester (78.2%, *n* = 219).

The mean meal consumption frequency of the study participants was 3.94 (SD, 1.04) times, and most (73.0%, *n* = 184) of them consumed meals four times or more daily during their current pregnancy. Regarding eating out per week, 85.6% (*n* = 238) ate out 0–4 times per week. More than half (64.6%, *n* = 181) of the participants consumed tea, cocoa, or coffee. Almost all the respondents (99.3%, *n =* 278) did not use alcohol in the previous seven days during their current pregnancy. Most of the women consumed processed foods five or more times per week (84.2%, *n =* 235), and a majority (85.6%, *n =* 238) of them were using iron and folic acid supplementation during their current pregnancy. Most participants (61.5%, *n =* 169) avoided certain foods; of them, 30.2% avoided unhealthy foods. More than half of the women were receiving information about nutrients during the pregnancy (63.3%, *n =* 150).

Regarding maternal knowledge, 59.1% (*n =* 165) and 87.1% (*n =* 243) of the participants were considered knowledgeable about the signs and symptoms of anemia and food sources that increase blood iron levels, respectively. The mean FDSK-11 score of the study participants was 8.54 (SD, 1.80). About half (46.4%) of the participants had low FDSK-11 scores (Table 2).

**Figure 1:**
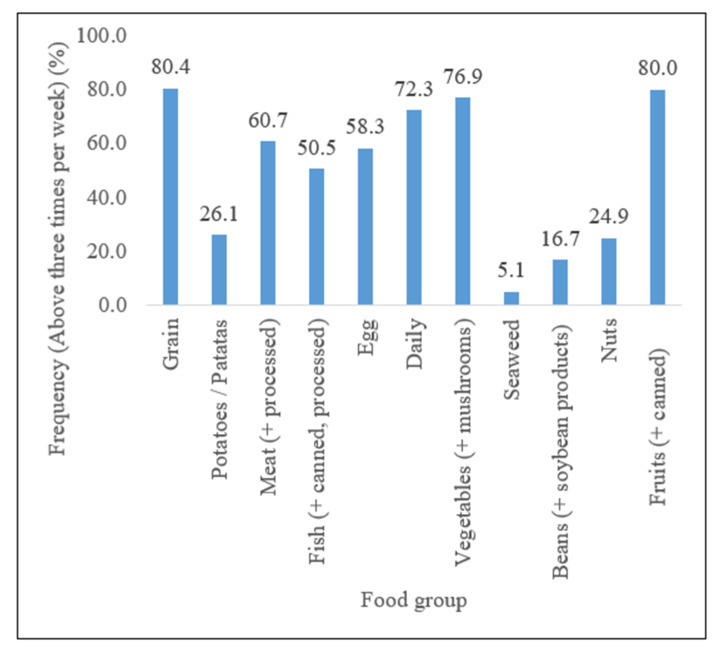
Food groups consumed by the participants over a week in the previous six months.

Figure 1 demonstrates the proportion of nutrients, by food group, consumed by the participants. In the week prior to the survey, 225 (80.4%) participants had consumed grain and 224 (80.0%) had consumed fruit. Furthermore, 213 (76.9%) reported the consumption of vegetables and mushrooms, and 201 respondents (72.3%) reported the consumption of dairy products including milk, cheese, and yogurt. Additionally, 170 (60.7%) participants consumed meat, and more than half (58.3%) of the respondents reported consuming eggs.

Approximately half (50.5%) of the respondents reported consuming fish, including canned and processed fish. Less than a third of the respondents consumed potatoes/*patatas*, nuts, beans (plus soybean products), and seaweed (26.1%, 24.9%, 16.7%, and 5.1%, respectively).

In the logistic regression analysis, poor maternal knowledge of food sources that increase blood iron levels (odds ratio (OR), 4.25; 95% CI, 1.47–12.32; *p* = 0.01) and less frequent consumption of meals (OR, 2.15; 95% CI, 1.08–4.29; *p* = 0.03) were significantly associated with poor dietary diversity (Table 3).

## 4. Discussion

This study analyzed factors associated with dietary diversity and sought to assess the quality of dietary intake of pregnant women in the Philippines from the results. The main findings of this study were as follows: (1) half of the women had a low dietary diversity during pregnancy; (2) more than half of the women avoided certain foods during pregnancy, mainly because they wanted to avoid unhealthy foods; (3) less than 30% of the respondents consumed beans; and (4) maternal knowledge of nutritional sources as well as frequent meal consumption during pregnancy were significantly associated with dietary diversity.

It has been previously reported that more than 50% of the pregnant women in the Philippines do not get sufficient nutrition [15]. Another study conducted in Ethiopia reported that high dietary diversity was prevalent in 45% of pregnant women [32]. In the current study, 46.4% of the participants had a low dietary diversity score, which is similar to the findings of the abovementioned studies. However, our findings showed a lower score than the minimum dietary diversity score of 57.9% reported from Indonesia [33]. From the above, it was found that pregnant women in the Philippines cannot eat nutritious meals throughout their pregnancy. This may be due to the lack of nutritional knowledge of pregnant women as shown in this study.

Regarding food avoidance, many (61.5%) of the participants in this study avoided at least one food during their current pregnancy. The major causes for avoiding certain foods were not because of taste or cultural reasons but to avoid unhealthy eating, to be a healthy mother, and to give birth to a healthy baby. The prevalence of food avoidance among the pregnant women in this study is in agreement with that reported in a study in northeastern Ethiopia [34]; however, another study in other parts of Ethiopia reported that only 27.3% of pregnant women avoided some foods during pregnancy [35]. This discrepancy might have been a result of cultural, social, economic, and environmental determinants of healthy dietary choices during pregnancy.

In the current study, most of the participants consumed grains and fruits. Furthermore, most of the women consumed vegetables, but only one-quarter of them consumed potatoes, which is a starchy vegetable. Over half of the women consumed animal products, but less than one-third of the women had a diet of beans. This finding is consistent with the findings of other studies conducted in the Philippines. It has been reported that cereals, mainly white rice, constitute half of the daily food intake of Filipino adults and the vegetable protein intake is inadequate [36]. Another study also demonstrated that a low intake of vegetables, fruits, and dairy is common because of the rice-dominant diet with few nutrient-dense foods in the Philippines [37,38,39]. The results of this study showed that animal protein intake during pregnancy was inadequate, and that vegetable protein intake was very inadequate. Inadequate protein intake can lead to anemia in pregnant women. Hence, reduced vegetable protein intake leads to poor dietary diversity, which increases the risk of anemia. However, anemia does not always occur because it is affected by various individual factors.

This study showed that the knowledge of nutritional sources to prevent anemia was significantly associated with a variety of dietary intakes. This meant that pregnant women who were knowledgeable about food sources tended to consume a higher quality diet. This finding is consistent with that reported in an Ethiopian study which showed that dietary knowledge is significantly associated with the quality of dietary practices [40]. A study in the United States also demonstrated that knowledge regarding diet among pregnant women is a predictor of dietary habits [41]. Thus, women with good dietary knowledge tend to think more about good eating habits, which can help in maintaining a nutritious, balanced, and quality diet. Further, an adequate understanding regarding a balanced diet leads to optimal health not only during pregnancy but throughout a woman’s life.

The study further showed that pregnant women who consume four or more meals a day are more likely to eat a wider variety of meals, which indicates that dietary frequency is significantly associated with dietary diversity. This finding is similar to that of a study in Ethiopia [42]. It is important to understand that increasing the number of meals during pregnancy leads to a balanced diet and thus to a quality diet. There are reports that education level is important for dietary diversity during pregnancy [43]. In addition, the Filipinos are traditionally influenced by the Spanish food culture and eat one light meal between breakfast and lunch, and another between lunch and dinner. In the Philippines, the term merienda is used to refer to these light meals. Our results suggested that merienda not only increases the number of meals consumed but also provides an opportunity to eat fruits, meats, and vegetables instead of snacks such as sweets. Thus, it is considered that this habit allows the consumption of various foods, which reduces poor dietary diversity. This might be explained by the fact that increased meal intake increases the chance of consuming different types of food.

Our study has some limitations. First, this research was only conducted in a medical facility, and our findings may not apply to the general public. Second, the estimations of dietary diversity practice in this study may be inaccurate due to recall bias and self-reporting. Third, it was not possible to analyze each gestation period because the majority of pregnant women were in their third trimester of pregnancy. Finally, while other studies included factors considered to be related to dietary diversity, such as livestock ownership [44], participation in food shopping [45], and husband support [46,47], they were not examined in this study.

## 5. Conclusions

Nutritional insecurity and associated health problems remain important public health issues, primarily in low- and middle-income countries, such as the Philippines. This study showed that the quality of dietary intake in half of the Filipino women was low during pregnancy. In addition, lack of knowledge about nutritious food sources and reduced dietary intake were associated with low dietary quality. Thus, it is important to improve the quality of dietary intake during pregnancy and increase knowledge regarding healthy dietary sources for the prevention of anemia. Educating women only during their pregnancy is not enough to increase their knowledge on proper nutrition and diet. It may be important for women to be educated about the importance of diet from an early age. In addition to improving the knowledge of pregnant women, it is recommended to enhance dietary guidance during prenatal health examinations in order to improve dietary diversity during pregnancy.

## Figures and Tables

**Table 1:** Sociodemographic characteristics of the study participants (*n* = 280).

Variable	Number	Percentage
Age in years		
<25	85	30.3
25–34	143	51.1
≥35	52	18.6
Occupation		
Unemployed	177	64.4
Employed	98	35.6
Education		
Primary	5	1.8
Secondary incomplete	28	10.0
Above secondary	246	88.2
Income per month		
<9999 pesos	159	59.6
>10,000 pesos	108	40.4
Parity		
Primiparous	114	40.7
Multiparous	166	59.3
Gestation		
First trimester	19	6.8
Second trimester	42	15
Third trimester	219	78.2

**Table 2:** Dietary practice and maternal knowledge on nutrients in pregnant women (*n =* 280).

Variable	Number	Percentage
Meal frequency per day		
1–3 times	68	27.0
≥4 times	184	73.0
Eating out per week		
0–4 times	238	85.6
≥5 times	40	14.4
Tea, cocoa, or coffee use		
No	99	35.4
Yes	181	64.6
Alcohol use in the previous 7 days		
No	278	99.3
Yes	2	0.7
Processed food eaten per week		
≥5 times	235	84.2
0–4 times	44	15.8
Taking IFAS during the current pregnancy		
No	40	14.4
Yes	238	85.6
Avoidance of any food or diet in the current pregnancy		
Yes	169	61.5
No	106	38.5
Reasons for avoiding		
To avoid unhealthy food (fatty, salty, sweet, alcohol, caffeine)	35	30.2
Culture	7	6.0
To be a healthy mother	34	29.3
To deliver a healthy baby	33	28.5
Other (dislike, discomfort)	7	6.0
Information on nutrients during pregnancy		
No	87	36.7
Yes	150	63.3
Maternal knowledge of signs and symptoms of anemia		
Slightly knowledgeable	114	40.9
Highly knowledgeable	165	59.1
Maternal knowledge of food sources that increase the blood iron levels		
Slightly knowledgeable	36	12.9
Highly knowledgeable	243	87.1
FDSK-11		
Higher	150	53.6
Lower	130	46.4

IFAS, iron and folic acid supplementation; FDSK-11, 11-item Food Diversity Score Kyoto.

**Table 3:** Multivariable regression analyses of dietary diversity among the participants (*n =* 280).

Variables	FDSK-11		Univariable Analysis			Multivariable Analysis		
	High	Low						
			OR	95% CI	*p*	OR	95% CI	*p*
Age in years								
<24	43	42	1			1		
25–34	84	59	0.72	0.42–1.23	0.23	0.75	0.37–1.51	0.41
≥35	23	29	1.29	0.65–2.58	0.47	1.92	0.78–4.75	0.16
Occupation								
Unemployed	90	87	1.40	0.85–2.31	0.18	1.25	0.65–2.40	0.50
Employed	58	40	1			1		
Income per month								
<9999 pesos	78	81	1.63	0.99–2.68	0.05	1.39	0.76–2.54	0.28
≥10,000 pesos	66	42	1			1		
Parity								
Primiparous	58	56	1.20	0.74–1.94	0.45	1.44	0.77–2.69	0.25
Multiparous	92	74	1			1		
Information on nutrients during pregnancy								
No	45	42	1.19	0.70–2.02	0.52	0.94	0.50–1.77	0.85
Yes	84	66	1			1		
Maternal knowledge of signs and symptoms of anemia								
Slightly knowledgeable	78	73	1.187	0.74–1.92	0.482	1.04	0.56–1.91	0.91
Highly knowledgeable	71	57	1			1		
Maternal knowledge of food sources that increase blood iron levels								
Slightly knowledgeable	68	78	2.249	1.09–4.65	0.029 *	4.25	1.47–12.32	0.01 *
Highly knowledgeable	81	52	1			1		
Meal frequency per day								
1–3 times	30	39	1.76	1.01–3.06	0.045 *	2.15	1.08–4.29	0.03 *
≥4 times	119	91	1			1		
Eating out per week								
0–4 times	131	107	1			1		
≥5 times	18	22	1.50	0.76–2.93	0.241	1.899	0.78–4.65	0.16

Significant association at 95% CI, * Significant at *p* < 0.05; FDSK-11, 11-item Food Diversity Score Kyoto; CI, confidence interval; OR, odds ratio.

## Data Availability

The data presented in this study are available on request from the corresponding author.
